# Self-Assembled Nanostructures of Red Fluorescent Amphiphilic Block Copolymers as Both Imaging Probes and Drug Carriers

**DOI:** 10.3390/polym10101120

**Published:** 2018-10-10

**Authors:** Shuo Huang, Xin Wei, Mingfeng Wang

**Affiliations:** School of Chemical and Biomedical Engineering, Nanyang Technological University, 62 Nanyang Drive, Singapore 637459, Singapore; shuoh8811@gmail.com (S.H.); S180023@e.ntu.edu.sg (X.W.)

**Keywords:** fluorescence, polymer, self-assembly, bioimaging, drug delivery

## Abstract

We report a red-fluorescent drug delivery system formed by biodegradable and biocompatible amphiphilic A-B-A block copolymers. Each polymer consists of a red fluorescent dye covalently bonded in the middle of hydrophobic block (B) of polylactone, tethered at both ends with poly[(oligo ethylene glycol) methyl ether methacrylate] (POEGMA) as the hydrophilic block. Two types of polylactones, i.e., semicrystalline poly(ε-caprolactone) (PCL) and amorphous poly(δ-decalactone) (PDL), respectively, were incorporated as the hydrophobic segment in the block copolymers. Using transmission electron microscopy, we characterized the self-assembled nanostructures formed by these amphiphilic block copolymers in mixtures of water/tetrahydrofuran or water/dimethylformamide. All of these polymers remained highly fluorescent in water, although some extent of aggregation-induced fluorescence quenching was still observed. Among the three types of polymers presented here, the polymer (RPO-3) containing an amorphous block of PDL showed the highest drug-loading capacity and the largest extent of drug release in acidic media. RPO-3 micelles loaded with doxorubicin as a model of anticancer drug showed sustainable intracellular release and cytotoxicity against HeLa cells.

## 1. Introduction

Multifunctional self-assembled nanostructures such as micelles and vesicles formed by block polymers that contain both fluorescent and therapeutic agents are important for applications such as imaging-guided therapy or so-called theranostics [1,2,3,4,5,6,7,8,9,10,11]. One of the conventional approaches to this end is to encapsulate organic fluorophores and therapeutic agents such as anticancer drugs into the same or different compartments of block copolymer self-assemblies [12,13,14,15,16,17,18,19]. Challenges that these approaches often face, however, include aggregation-caused fluorescence quenching, photobleaching of organic chromophores, and leaching of the organic chromophores from the polymer matrices.

To address these challenges, we have explored a bioinspired strategy by covalently linking a single organic chromophore in the middle of a polymer chain such as poly(ε-caprolactone) (PCL), resembling the structure of fluorescent proteins existing in bioluminescent systems such as glowing jelly fish [20,21,22,23,24]. When the polymer chain is long enough, for example, with a number-average degree of polymerization over 100 on both sides of an organic chromophore, these polymers remain highly fluorescent and photostable in solid states. The polymer chains effectively segregate the organic chromophores from each other, thus preventing fluorescence quenching caused by intermolecular aggregation. Moreover, the hydrophobicity and the crystallinity of PCL inhibit the access of O_2_ and H_2_O to a chromophore in the middle of a polymer chain, which contributes to the photostability.

In order to disperse these hydrophobic PCL–dye–PCL polymers into water for applications such as bioimaging, we previously employed a method of nanoprecipitation, in which the PCL polymers were codissolved with a polymeric surfactant such as Pluronic F127 into a common good solvent such as tetrahydrofuran, followed by flash precipitation into a large excess of water under stirring and ultrasound sonication [21]. The organic solvent can be removed by either evaporation in air or dialysis against pure water. Although this method has been widely used to disperse hydrophobic molecules as nanoparticles into water, without extra modification of the surfactant or use of advanced techniques such as microfluidics [25,26,27,28,29,30], precise control of the particle size and shape is generally difficult. In addition, this nanoprecipitation process often results in some empty micelles formed by the surfactant molecules themselves, which requires extra efforts to remove them in order to increase the effective concentration of the encapsulated materials [31]. Furthermore, it has been reported that the crystallinity of PCL often results in low drug-loading capacity and retarded drug release [30,32].

**Scheme 1:**
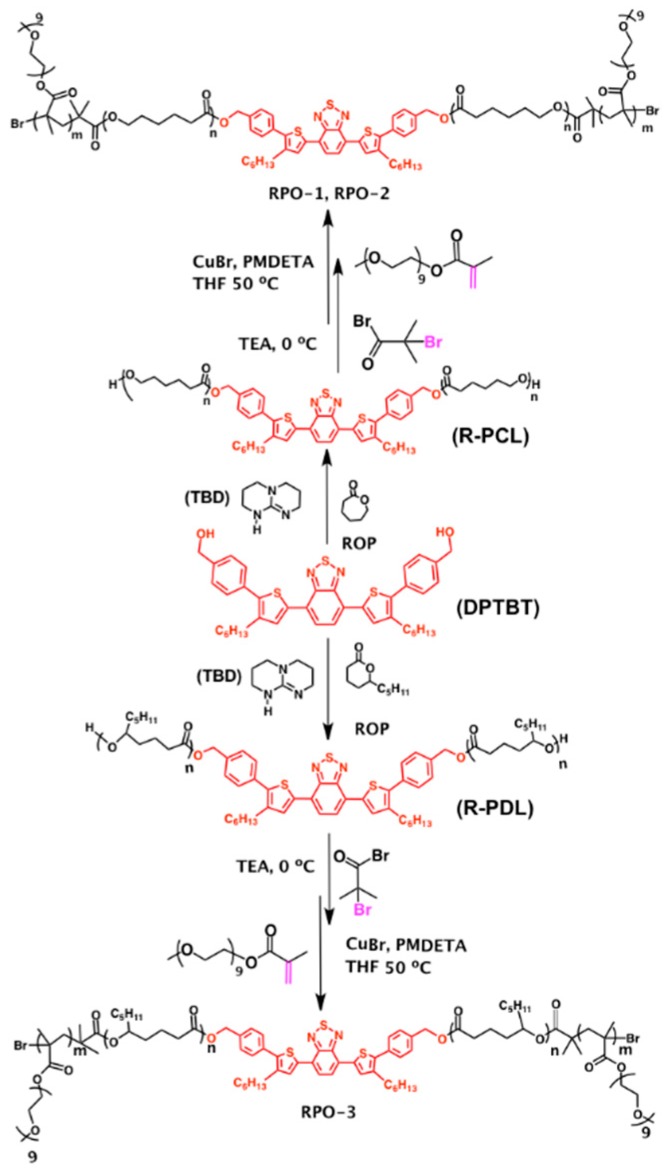
Schematic illustration of chemical structures and synthetic route to RPO-1, RPO-2, and RPO-3 polymers.

To address these issues in nanoprecipitation of hydrophobic PCL–dye–PCL polymers, on the one hand, herein we report our efforts of growing hydrophilic polymers, using poly[(oligo-ethylene-glycol) methyl ether methacrylate] (POEGMA) as an example, from both chain ends of a red fluorescent PCL–dye–PCL polymer. The resulting amphiphilic block copolymers (RPO-1–3, Scheme 1) are dispersible into water without use of additional surfactants. Moreover, the size and the morphology that these polymers self-assemble in water are controllable by tuning the solvent polarity and the hydrophobic/hydrophilic ratio of the block copolymer. On the other hand, we replaced the crystalline PCL block with an amorphous poly(δ-decalactone) (PDL). In addition, the monomer, δ-decalactone used for the synthesis of PDL, is a natural compound and an Food and Drug Administration (FDA) approved flavoring agent [32,33,34]. We examined how the crystallinity of the hydrophobic block (PCL vs. PDL) affect the self-assembled nanostructures in water, the drug-loading capacity using doxorubicin (DOX) as an example of an anticancer drug, and the controlled release in aqueous buffer with different pH values. Finally, we characterized the cellular internalization and cytotoxicity of DOX-loaded micelles of PDL-based polymer, RPO-3, using HeLa cells as a cancer model cell line.

## 2. Materials and Methods

### 2.1. Materials and Characterization

ε-Caprolactone (97%), δ-decalactone (≥98%), α-bromoisobutyryl bromide (BIBB) (98%), 1,5,7-triazabicyclo[4.4.0]dec-5-ene (TBD) (98%), oligo(ethylene glycol) methyl ether methacrylate (OEGMA, *M*_n_ = 300 g/mol) and all solvents were purchased from Sigma Aldrich (Saint Louis, MO, USA). (Oligo ethylene glycol) methyl ether methacrylate (OEGMA) monomer was passed through basic alumina column before use. Dulbecco’s Modified Eagle’s Medium (DMEM), fetal bovine serum (FBS), penicillin/streptomycin mixture, phosphate buffered saline (PBS) and PrestoBlue cell viability reagent was purchased from Life Technologies (Singapore). Other reagents including 4′, 6-diamidino-2-phenylindole (DAPI) and formalin solution were ordered from Sigma-Aldrich (Singapore). All reactions were carried out under N_2_ atmosphere unless noted specifically.

^1^H-NMR spectra were recorded on a Bruker AV 300 nuclear magnetic resonance (NMR) spectrometer (Rheinstetten, Germany) using tetramethylsilane as an internal standard at 25 °C. The number average molecular weight (*M*_w_) and molecular weight distribution (*M*_w_/*M*_n_) were measured by gel permeation chromatography (GPC) system (Agilent 1260, Agilent Technologies, Santa Clara, CA, USA) equipped with waters 1260 pump and an Agilent 1260 refractive index detector (Agilent Technologies, Santa Clara, CA, USA) and a styragel column (Agilent Technologies, Santa Clara, CA, USA). Tetrahydrofuran (THF) was used as the eluent (1 mL/min), and polystyrene was used as the standard for calibration. Transmission electron microscope (TEM) measurements were carried out on a Carl Zeiss Libra 120 (Carl Zeiss Microscopy GmbH, Oberkochen, Germany) Plus electron microscope operating at an acceleration voltage of 120 kV. Ultraviolet–visible (UV–vis) absorption spectra were recorded on a UV-2450 (SHIMADZU, Kyoto, Japan) spectrophotometer. Steady-state photoluminescence emission spectra were recorded with LS 55 (PerkinElmer, Waltham, MA, USA) fluorescence spectrometer. The particle size was measured with a dynamic light scattering (DLS) instrument, Zetasizer Nano ZS (Malvern, UK).

### 2.2. Synthesis of Red Fluorescent Homopolymers: Polycaprolactone (R-PCL) and Polyedecalactone (R-PDL)

The synthesis of the fluorescent initiator di-phenyl thiophene benzothiadiazole (DPTBT) followed the procedure reported previously [20].

The monomer, either ε-caprolactone (620 mg, 5.4 mmol) or δ-decalactone (1.14 g, 6.7 mmol) was transferred into a flask containing the initiator and continuously stirred for 10–15 min to make a homogeneous mixture. TBD was dissolved in chloroform then added under a nitrogen atmosphere and the mixture was allowed to react for 12 h at 45 °C. The viscous liquid obtained was subsequently quenched by adding the acetic acid solution. The polymer was precipitated in cold methanol twice. The dried R-PCL was obtained as a red powder with a yield of 70%, and R-PDL was obtained as a red viscous liquid with a yield of 77%.

### 2.3. Synthesis of Red-Fluorescent Amphiphilic Block Copolymers (RPO-1–3)

Preparation of macroinitiators end-capped by isobutyryl bromides: Typically, red-PCL or red-PDL (0.1 mmol) end-capped by –OH groups was dissolved in anhydrous tetrahydrofuran (THF) (20 mL), and then triethyl amine (20 mmol) was added under an argon atmosphere at 0 °C. After 0.5 h, the BIBB (20 mmol) was added dropwise to the mixture under magnetic stirring. The reaction was carried out at 0 °C for 2 h and then stirred for 24 h at room temperature. Then the mixture was precipitated in diethyl methanol twice and washed by water and methanol. After being drying under vacuum, polymers end-capped with isobutyryl bromides were obtained with a yield of 70%.

Synthesis of red-fluorescent amphiphilic block copolymers (POEGMA-*b*-PCL–dye–PCL-*b*-POEGMA, and POGEMA-*b*-PDL-dye-PDL-*b*-POEGMA): The macroinitiator prepared above (R-PCL_108_-Br, R-PCL_82_-Br, or R-PDL_42_-Br) (0.02 mmol), CuBr (0.04 mmol), and OEGMA (*M*_n_ = 300, 2.5 mmol) were dissolved in 0.6 mL of THF in a 25 mL two-neck flask, and the solution was degassed with three freeze-pump-thaw cycles. Then *N*,*N*,*N*′,*N*″,*N*″-pentamethyldiethylenetriamine (PMDETA) (8.0 µL, 0.04 mmol) was injected into the above solution, and the mixture was degassed with two freeze-pump-thaw cycles. The polymerization was carried out at 50 °C for 2.5 h. The reaction mixture was diluted with THF and then passed through a short neutral Al_2_O_3_ column to remove the copper catalyst. The resulting solution was concentrated and poured into hexane to precipitate the products. The final polymer products were filtered and dried under vacuum at room temperature. The yields of the polymers and the molecular weights measured with gel permeation chromatography (GPC) are summarized in Table 1.

### 2.4. Preparation of RPO Micellar Structures

Method 1: 4 mL di-water was slowly dropped (30 mL/h) into a solution of a block copolymer (25 mg/mL) in THF under stirring. And then the mixture was immediately dialyzed against water for 48 h to remove the organic solvent. During the dialysis process, water will be changed every 12 h.

Method 2: 4 mL of di-water was slowly dropped (15 mL/h) into a solution of a block copolymer (10 mg/mL) in THF under stirring. The remaining procedure was the same as Method 1.

Method 3: 4 mL di-water was slowly dropped (15 mL/h) into a solution of a block copolymer (20 mg/mL) in DMF under stirring. The remaining procedure was the same as Method 1.

Method 4: 4 mL di-water was slowly dropped (15 mL/h) into a solution of a block copolymer (20 mg/mL) in DMF under stirring. And then the mixture stood overnight before dialysis against water for 48 h to remove the organic solvent. The remaining procedure was the same as Method 1.

### 2.5. Encapsulation of Doxorubicin (DOX) into RPO-3 Nanoparticles (NPs)

The strategy is similar to Method 3. Briefly, 4 mL di-water was slowly dropped (15 mL/h) into a solution of RPO-3 (20 mg) and DOX (2 mg) in 1 mL of DMF under stirring. Then the mixture was immediately dialyzed against water for 48 h to remove organic solvent. During dialysis process, water was changed every 12 h.

### 2.6. In Vitro DOX Release with Near-Infrared (NIR) Laser Irradiation

In vitro release profiles of DOX were evaluated by the dialysis method. First, a dialysis bag (MWCO 3500, SPECTRUM, Fort Worth, TX, USA) was filled with 1 mL of DOX@RPO-3 NPs soaked in a tube containing 40 mL of PBS 7.4 or 5.0 at 37 °C incubator. At predetermined time intervals, 3 mL of the buffer was withdrawn and it was replaced with 3 mL of fresh PBS 7.4 or 5.0. Then a release concentration of free DOX was calculated based on a calibration curve by fluorescence intensity with an excitation wavelength at 495 nm and the emission wavelength at 550 nm.

### 2.7. In Vitro Cellular Uptake of RPO-3 Micelles and DOX@RPO-3 Composite Micelles

HeLa cells were seeded into a 12-well plate and cultured in 37 °C with 5% CO_2_ in DMEM medium supplemented with 10% FBS and 1% penicillin-streptomycin. After 12 h for attachment, blank RPO-3 NPs and DOX@RPO-3 NPs (final DOX concentration: 25 μg mL^−1^) were added to the medium and incubated with HeLa cells. After 2 h or 12 h incubation, the cell culture medium was removed and each well was washed with 1 × PBS for five times. Cells were fixed by formalin solution for 30 min and then washed by 1 × PBS extensively for three times. Cell nucleus was stained by DAPI for 10 min at room temperature and then the samples were washed three times and then added in fresh 1 × PBS. Lasers of 405 and 488 nm were used to excite DAPI and DOX@RPO-3 respectively. The corresponding fluorescence emissions were recorded by a confocal laser scanning microscopy (LSM 710, Carl Zeiss Microscopy GmbH, Oberkochen, Germany) using a band-pass filter combination including 410–507, 495–550, and 600–700 nm for imaging in three individual channels (Objective: EC Plan-Neofluar 20×/0.30 M27; dimension is 1024 × 1024).

### 2.8. Cytotoxicity of RPO-3 and DOX@RPO-3 NPs

The cytotoxicity of blank RPO-3 NPs and DOX@RPO-3 NPs in HeLa cells were studied using a similar procedure reported previously [35,36,37].

## 3. Results and Discussion

### 3.1. Polymer Synthesis and Structural Characterization

The synthetic routes to the two types of amphiphilic block copolymers are shown in Scheme 1. The synthesis started from ring-opening polymerization of ε-caprolactone or δ-decalactone from benzyl alcohols of a red-fluorescent dye, DPTBT, as the initiator, and an organic base TBD as the catalyst [38], resulting in homopolymers (R-PCL or R-PDL) with a single fluorophore in the middle of each polymer chain. Compared to the polymerization route that we reported previously using Sn(II) 2-ethylhexanoate as the catalyst which is highly toxic [20], the expected low toxicity of TBD used in the present study may benefit the biomedical applications of the synthesized polymers, particularly for drug delivery and tissue engineering.

In order to introduce hydrophilic polymers into the intrinsically hydrophobic R-PCL or R-PDL homopolymers, the -OH end-capping groups from the chain ends of these homopolymers were first reacted with BIBB to introduce the initiating sites for the following step of atom transfer radical polymerization (ATRP) of OEGMA as a hydrophilic monomer. Both RPO-1 and RPO-2 contain a hydrophobic and semicrystalline block of PCL, but with different chain lengths. By contrast with RPO-1–2, RPO-3 contains a hydrophobic and amorphous block of PDL [32,34]. Alexander and coworkers have reported that the micelles formed by linear amphiphilic diblock copolymer mPEG-*b*-PDL showed seven-fold higher drug-loading capacity than those formed by mPEG-*b*-PCL with similar molecular weight [32]. We characterized the chemical structures of RPO-(1–3) using both ^1^H-NMR (Figures S1 and S2) and GPC (Figure S3a–c), and the results are summarized in Table 1.

### 3.2. Self-Assembly of RPO-(1–3) in Aqueous Solutions

**Figure 1:**
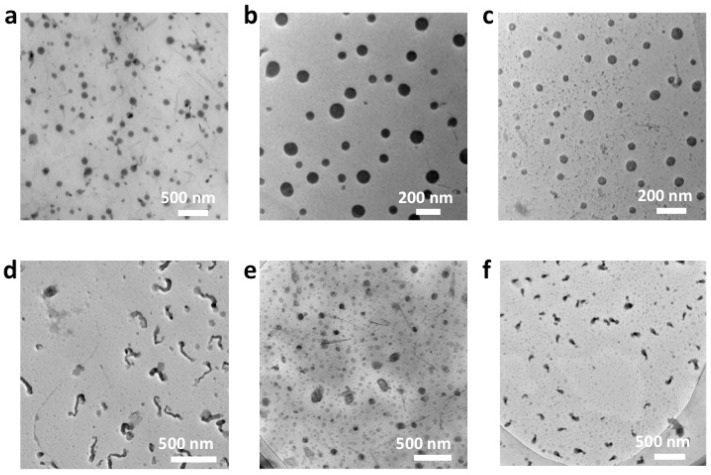
Representative transmission electron microscope (TEM) images of (**a**) RPO-1 prepared by Method 1, (**b**) RPO-1 prepared by Method 2, (**c**) RPO-1 prepared by Method 3, (**d**) RPO-1 prepared by Method 4, (**e**) RPO-2 prepared by Method 4 and (**f**) RPO-3 prepared by Method 4.

We next investigated the morphology of the nanoparticles (NPs) formed by self-assembly of RPO-(1–3) polymers without additional surfactants. The self-assembly was induced by slow addition distilled water which is a selective solvent for POEGMA, into a polymer solution dissolved in either THF or DMF as common good solvents due to their good miscibility with water. More details of the sample preparation are described in Section 2.4. We carried out a preliminary screening of different experimental conditions, including solvent polarity, addition speed of water, and the polymer concentration, in order to understand how these factors affect the morphology of the self-assemblies. A representative TEM image (Figure 1a) of RPO-1 NPs prepared by Method 1 (Section 2.4) shows a mixture of dark spheres, needles and ribbons with relatively low electron density. The details of each assembly can be seen more clearly in the high-magnification TEM image shown in Figure S4, which suggest that each dark sphere might correspond to a large compound micelle as previously observed by Eisenberg and coworkers [39,40]. When both the concentration of RPO-1 and the water-addition rate decreased (Method 2, Section 2.4), the formation of rod-like micelles was suppressed, resulting in a major population of large compound micelles with varying sizes from tens up to 100 nm (Figure 1b). When DMF was chosen as the good solvent (Method 3, Section 2.4), one can observe loosely packed large compound micelles with irregular surfaces and obvious presence of individual micelles with smaller sizes of a few nanometers (Figure 1c and Figure S5). When the DMF/water mixture of RPO-1 prepared in the same way as Method 3 but stood overnight to reach the equilibrium before being quenched by excess water and the following dialysis, the TEM image of the sample prepared this way (so-called Method 4, the details of which are described in Section 2.4) shows a unique morphology of short worm-like structures (Figure 1d and Figure S6), with the presence of some smaller spherical particles as well.

Compared to RPO-1, RPO-2 with shorter block lengths of both POEGMA and PCL formed micellar structures with different morphologies. For instance, the TEM image (Figure 1e and Figure S7) of RPO-2 micelles prepared by Method 4 shows a mixture of spheres, rods and ribbons, similar to that of RPO-1 prepared by Method 1.

We also used Method 4 to prepare the micelles of RPO-3 with an amorphous hydrophobic block of PDL. The TEM image (Figure 1f and Figure S8) shows a mixture of tadpole-like micelles and spherical micelles with smaller sizes. At the same time, some spherical large-compound micelles could also be observed in some regions. (Figure S8) These results suggest that the self-assembly of these amphiphilic triblock copolymers is sensitive to both the chemical structure and the crystallinity of the hydrophobic block. Nevertheless, further efforts are needed in the future to understand the kinetics and thermodynamics in the self-assembly process in order to prepare micelles or other structures of self-assemblies with uniform sizes and morphologies, for example, by using block copolymers with narrower polydispersities.

### 3.3. Optical Properties of RPO-(1–3) and Their Self-Assembled Nanostructures in Water

**Figure 2:**
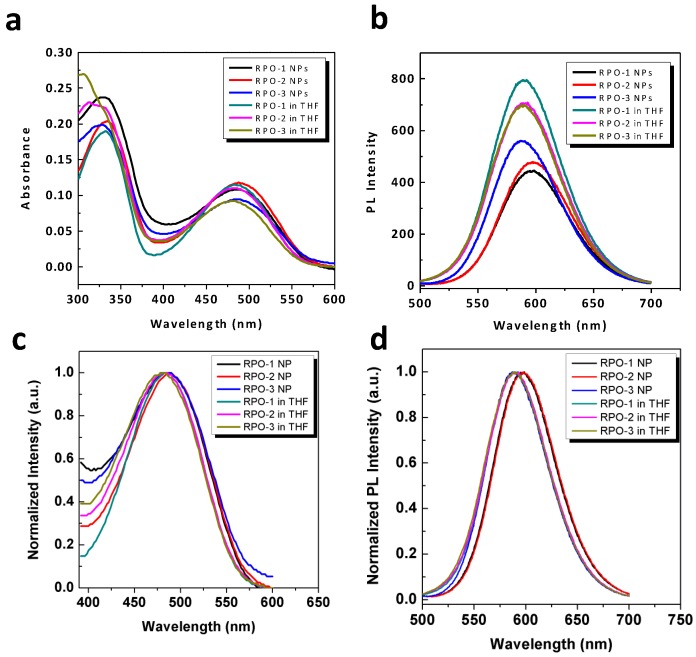
(**a**) Ultraviolet–visible (UV–vis) absorption spectra and (**b**) emission spectra of three polymers at the same concentration in THF and water. (**c**) Normalized UV–vis absorption spectra and (**d**) emission spectra.

We characterized the optical properties of the nanostructures formed by RPO-(1–3) in water through Method 1 as an example using both UV–vis absorption spectroscopy and fluorescence spectroscopy. There is no significant difference in the absorption spectra among the three polymers in THF and their nanoparticles suspended in water (Figure 2a,c), which suggests no obvious intra-molecular interaction of organic dye. In contrast to the little change in the absorption spectra, the fluorescence emission peaks of both RPO-2 and RPO-3 nanoparticles in the normalized spectra (Figure 2d) show a slight but obvious redshift compared to the aggregation-free polymers dissolved in THF. The extent of such a redshift is independent of the polymer chain length and the crystallinity of the hydrophobic block. In addition, there is a general decrease of the fluorescence intensity for each type of the polymers in the form of NPs in water versus the same polymer with matched optical density in THF, as shown in the non-normalized fluorescence emission spectra (Figure 2b). These results suggest some extent of fluorescence quenching mainly induced by the π-π stacking between DPTBT chromophores inside the hydrophobic domains of the nanostructures formed by these amphiphilic block copolymers in water. Nevertheless, further quantitative analysis of the relationship between the polymer structure and the fluorescence quenching is difficult, given the relatively large polydispersity of the micellar size and morphology under the present experimental conditions.

### 3.4. Encapsulation of Anticancer Drugs into RPO Micellar Structures and Controlled Release

**Figure 3:**
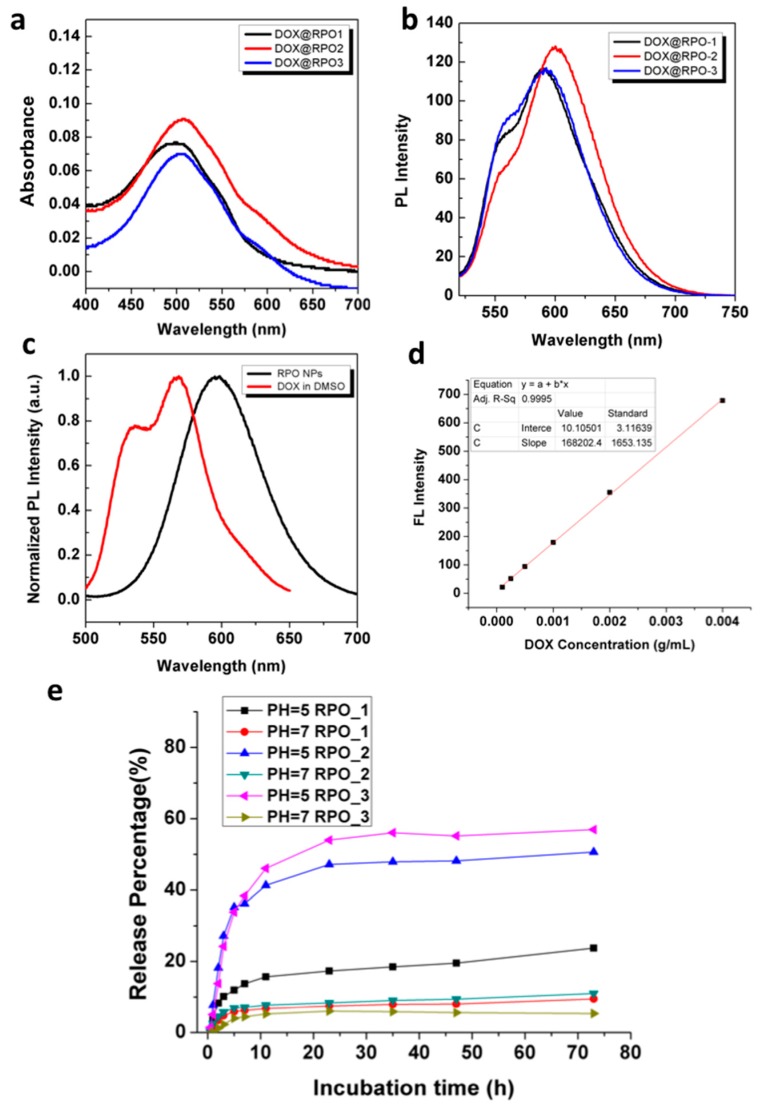
(**a**,**b**) UV–vis absorption (**a**) and fluorescence emission (**b**) spectra of RPO polymers with doxorubicin (DOX) encapsulated. (**c**) Fluorescence emission spectra of DOX in DMSO and RPO nanoparticles (NPs) in water excited at 488 nm. (**d**) Calibration curve of DOX concentration vs. fluorescence intensity at 550 nm. (**e**) Release profile of DOX@RPO within 3 days in pH = 5.0 or 7.4 aqueous solution incubated at 37 °C (C_DOX_ = 50 µg mL^−1^).

In this section, we studied whether the self-assembled structures formed by the RPO polymers described above could serve as drug carriers, particularly by encapsulating hydrophobic anticancer drugs in the hydrophobic domains of the polymer self-assemblies. We also tried to understand how the drug-loading capacity and drug release kinetics are related to the polymer structures. Specifically, we chose DOX as a model of anticancer drug, considering the little overlap between the fluorescence emission spectrum of DOX and that of the RPO polymers (Figure 3b,c), despite the significant overlap in their absorption spectra (Figure 3a). DMF was chosen as a common good solvent for both RPO polymers and DOX. The details of encapsulating DOX into the RPO carriers are described in Section 2.5.

The amount of DOX encapsulated in each micelle formulation was calculated using the fluorescence intensity at 550 nm of nanoparticles diluted by DMSO and a calibration curve of DOX of various concentrations in DMSO (Figure 3d). The RPO-3 polymer containing an amorphous hydrophobic block of PDL gave a DOX-loading content of 7% by weight. This value is obviously higher than that of RPO-1 (4%) and RPO-2 (3%), both of which contain a semicrystalline hydrophobic block of PCL. This result was expected and consistent with what was reported previously by Alexander’s group [32].

Figure 3e shows the in vitro drug release profiles of DOX@RPO micelles at two pH values (i.e., pH = 5.0 and pH = 7.4). At pH 7.4, the micelles formed by each of the three polymers (RPO-(1–3)) showed comparable minimal release (<10%) of DOX. In contrast to pH 7.4, a dramatic difference of the release kinetics was observed at pH 5.0. Specifically, the cumulative release of DOX from DOX@RPO-1 only reached ca. 20% after being incubated in the pH 5.0 buffer over 72 h, which is much lower compared to that (ca. 50%) of DOX@RPO-2 and DOX@RPO-3 which were incubated under the same condition as that for DOX@RPO-1. And the extent of the release of DOX from DOX@RPO-3 is slightly higher than that from DOX@RPO-2 during the same incubation period (i.e., 24–72 h).

The results above about the drug loading and controlled release indicate that an amorphous hydrophobic block of PDL, compared to a semicrystalline hydrophobic block of PCL, in the amphiphilic block copolymers not only gives higher drug-loading capacity, but also promotes the drug release [41]. In addition, shorter PCL blocks benefits faster and a higher extent of drug release.

### 3.5. Cellular Internalization of DOX@RPO-3 Nanoparticles and Cytotoxicity

**Figure 4:**
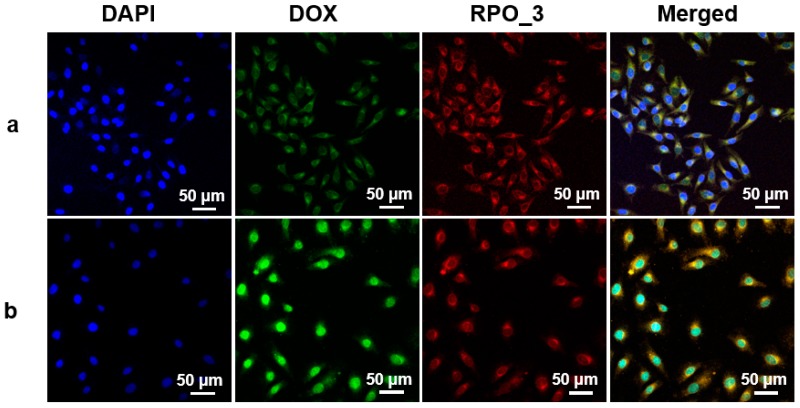
Fluorescence images of HeLa cells after being incubated with DOX@RPO-3 NPs over different (**a**) after 2 h incubation and (**b**) DOX@RPO-3 NPs after 12 h incubation, respectively. The fluorescence of 4′, 6-diamidino-2-phenylindole (DAPI) (λ_ex_ = 405 nm), DOX (λ_ex_ = 488 nm, λ_em_ = 495–550 nm), and RPO-3 (λ_ex_ = 488 nm, λ_em_ = 600–700 nm) was pseudo labeled with blue, green, and red, respectively.

Considering the superior performance of DOX@RPO-3 in the drug-loading capacity and controlled release as described above, we only chose DOX@RPO-3 as an example in the following cellular study which includes imaging the cellular internalization and cytotoxicity. The cellular uptake behavior was further evaluated by confocal laser scanning microscopy (CLSM). The blank RPO-3 NPs can be internalized into the cytoplasm of HeLa cells after 2-h incubation (Figure S9, Supporting Information). Due to the slight overlap of emission peak of DOX and RPO-3 NPs under 488-nm excitation, the bandpass filter was used to separate their emission signal, which was 495–550 nm for DOX and 600–700 nm for RPO-3 NPs, respectively. It can be clearly observed that DOX is accumulated around nuclei after 2-h incubation (Figure 4a), which is completely overlapped with the fluorescence signal from RPO-3. Further increase of the incubation time to 12 h resulted in more release of the DOX from the micelles and significant accumulation of DOX in nuclei of HeLa cells (Figure 4b), while the majority of RPO-3 carriers remained in the cytoplasm. Such sustainable release behavior, which is similar to that observed in the in vitro release profile, is important to minimize the undesired drug leaching and side effects of toxicity.

**Figure 5:**
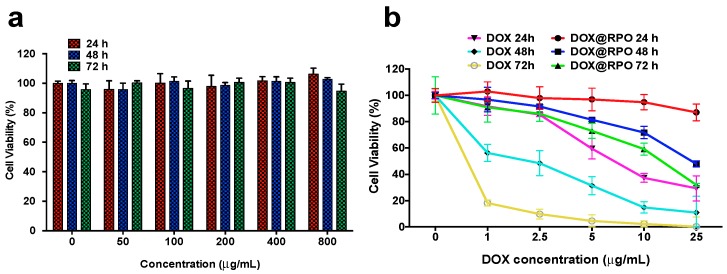
(**a**) Cytotoxicity of HeLa cells tested by PrestoBlue assay after treatments with various concentrations of RPO-3 micelles for 24, 48, and 72 h, respectively. (**b**) Cell viability of HeLa cells tested by PrestoBlue assay after treatments with various DOX concentrations of DOX@RPO-3 NPs and free DOX for 24, 48, and 72 h, respectively. Data were shown as means ± SD (*n* = 5).

Finally, we investigated the cytotoxicity of blank RPO-3 micelles and DOX@RPO-3 micelles versus carrier-free DOX·HCl. Figure 5a shows that no obvious toxicity was observed in HeLa cells, which were incubated with blank RPO-3 micelles at a concentration up to 800 µg mL^−1^. Figure 5b shows the cytotoxicity of the DOX@RPO-3 micelles with a range of effective concentrations of DOX, using carrier-free DOX·HCl with the same concentration as the control. Both the incubation time and the concentration of DOX affect the cytotoxicity. In general, DOX@RPO-3 micelles showed obviously lower cytotoxicity than carrier-free DOX·HCl under the same experimental conditions, which is consistent with the sustainable release of DOX as observed in Figure 3e and Figure 4. Similar trends were observed in our previously reported polymeric drug carrier systems [30,35,36,37]. When the incubation time was kept at 24 h, DOX@RPO-3 micelles showed minimal cytotoxicity against HeLa cells in the range of DOX concentration up to 25 µg mL^−1^. Nevertheless, ca. 60% HeLa cells were killed when the incubation time with DOX@RPO-3 micelles (containing 25 µg mL^−1^ of DOX) was extended over 72 h. Again, this result is consistent with the relatively slow release and accumulation of DOX in HeLa cells as observed in Figure 4.

Overall, the results above demonstrate that RPO-3 micelles can serve as an effective carrier for hydrophobic drugs for improved drug delivery and controlled release.

## 4. Conclusions

We have presented a dual functional anticancer drug delivery system formed by fluorescent and biodegradable amphiphilic block copolymers (denoted as RPO-(1–3)). Each polymer consists of POEGMA-*b*-polylactone-*b*-POEGMA with a red fluorescent dye covalently bonded in the middle of a hydrophobic polylactone block. Two types of polylactones, i.e., semicrystalline poly(ε-caprolactone) (PCL) and amorphous poly(δ-decalactone) (PDL), respectively, were incorporated as the hydrophobic segment in the block copolymers. We used TEM to study the effects of the crystallinity and the chain length of the polylactone block on the morphologies of the self-assemblies formed by these amphiphilic block copolymers in mixtures of water/THF or water/DMF. All of these block copolymers remained highly fluorescent in water, thanks to the segregation and protection effect of the polylactone block, although some extent (ca. 20–50%) of fluorescence quenching was still observed in water compared to the aggregation-free state in THF. We further demonstrated that hydrophobic drugs such as DOX could be encapsulated into the hydrophobic domains of RPO micelles. Among the three block copolymers synthesized here, RPO-3 containing an amorphous block of PDL showed the highest drug-loading content up to 7% by weight and the largest extent of drug release in an acidic environment at pH 5.0. DOX-loaded RPO-3 micelles could be efficiently internalized by HeLa cells and showed sustainable intracellular drug release and cytotoxicity. We believe that these results will provide a useful guideline to design and optimize fluorescent polymeric drug carriers for potential applications such as imaging-guided therapy, so-called theranostics, in the future.

## Figures and Tables

**Table 1:** Summary of polymer characterizations.

Entry No.	Polymer Structure	Yield	*M*_n_^1^	*M*_w_/*M*_n_
R-PCL-1	PCL_54_-dye-PCL_54_	70%	13,000	1.5
R-PCL-2	PCL_41_-dye-PCL_41_	68%	10,000	1.7
R-PDL	PDL_22_-dye-PDL_22_	77%	8,000	1.7
RPO-1	POEGMA_62_-*b*-PCL_54_-dye-PCL_54_-*b*-POEGMA_62_	71%	50,000	1.9
RPO-2	POEGMA_17_-*b*-PCL_41_-dye-PCL_41_-*b*-POEGMA_17_	67%	20,000	2.1
RPO-3	POEGMA_25_-*b*-PDL_22_-dye-PDL_22_-*b*-POEGMA_25_	40%	23,000	3.3

^1^ Molecular weight was calculated from gel permeation chromatography (GPC) running in tetrahydrofuran (THF) as eluent.

## Supplementary Materials

The following are available online at http://www.mdpi.com/2073-4360/10/10/1120/s1. Figure S1: (a) ^1^H-NMR (300 MHz, CDCl_3_) spectra of homopolymer Red-PCL and (b) Red-PDL. Figure S2. (a) ^1^H-NMR (300 MHz, CDCl_3_) spectra of amphiphilic block copolymer RPO-1, (b) RPO-2 (b) and (c) RPO-3. Figure S3: (a) GPC traces of RPO-1, (b) RPO-2 and (c) RPO-3. Figure S4: A representative high-magnification TEM image of RPO-1 micellar structures prepared by Method 1. Figure S5: A representative high-magnification TEM image of RPO-1 micellar structures prepared by Method 3. Figure S6: A representative high-magnification TEM image of RPO-1 self-assemblies prepared by Method 4. Figure S7. A representative high-magnification TEM image of RPO-2 self-assemblies prepared by Method 4. Figure S8. Representative low-magnification (a) and high-magnification (b) TEM images of RPO-3 self-assemblies prepared by Method 4. Figure S9. Fluorescence images of HeLa cells after being incubated with blank RPO-3 micelles over 2 h.
