# Supplementary material for: Self-Assembled Nanostructures of Red Fluorescent Amphiphilic Block Copolymers as Both Imaging Probes and Drug Carriers
Source: Polymers (Basel). 2018 Oct 10;10(10):1120. doi: 10.3390/polym10101120 (PMC6403604; doi:10.3390/polym10101120)
Supplement: Supplementary file 1 [file polymers-10-01120-s001.pdf]

Supplementary Materials: Self-Assembled Nanostructures of Red Fluorescent Amphiphilic Block Copolymers as Both Imaging Probes and Drug Carriers

Shuo Huang, Xin Wei and Mingfeng Wang

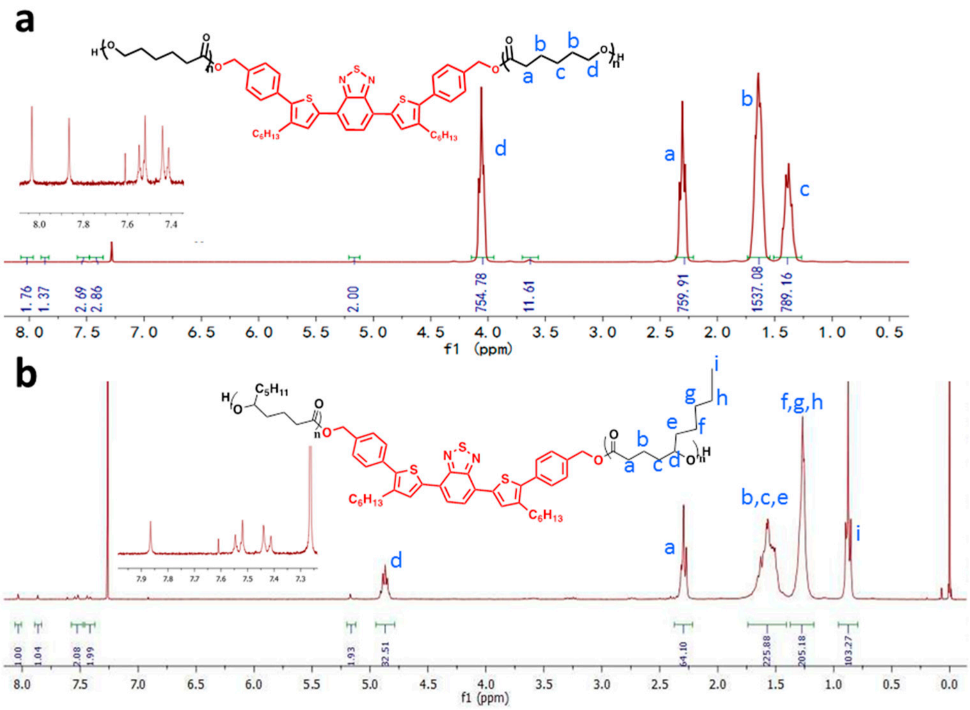

Figure S1. (**a**) ^{1}H-NMR (300 MHz, $CDCl_3$) spectra of homopolymer R-PCL and (**b**) R-PDL.

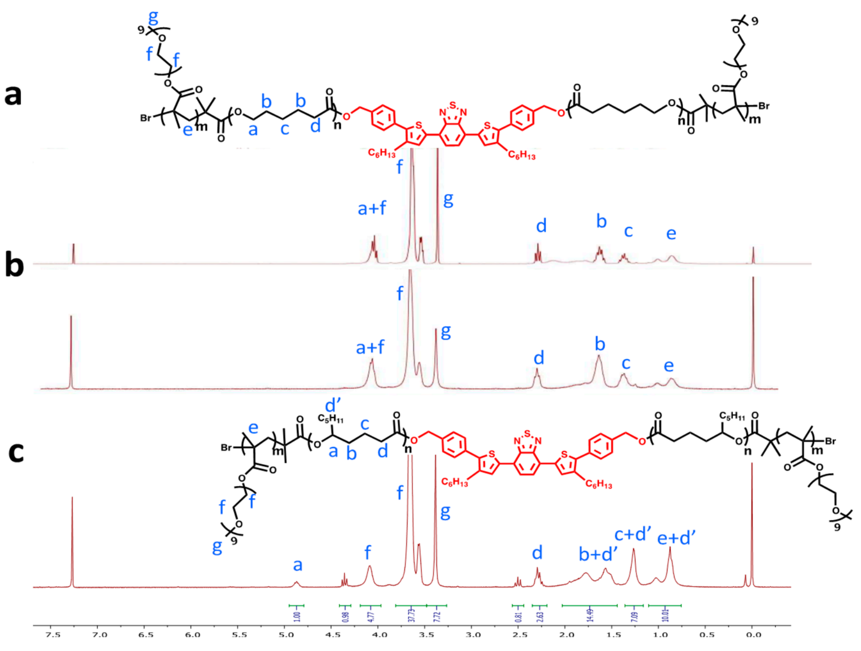

Figure S2. (**a**) ^{1}H-NMR (300 MHz, $CDCl_3$) spectra of amphiphilic block copolymer RPO-1; (**b**) RPO-2 and (**c**) RPO-3.

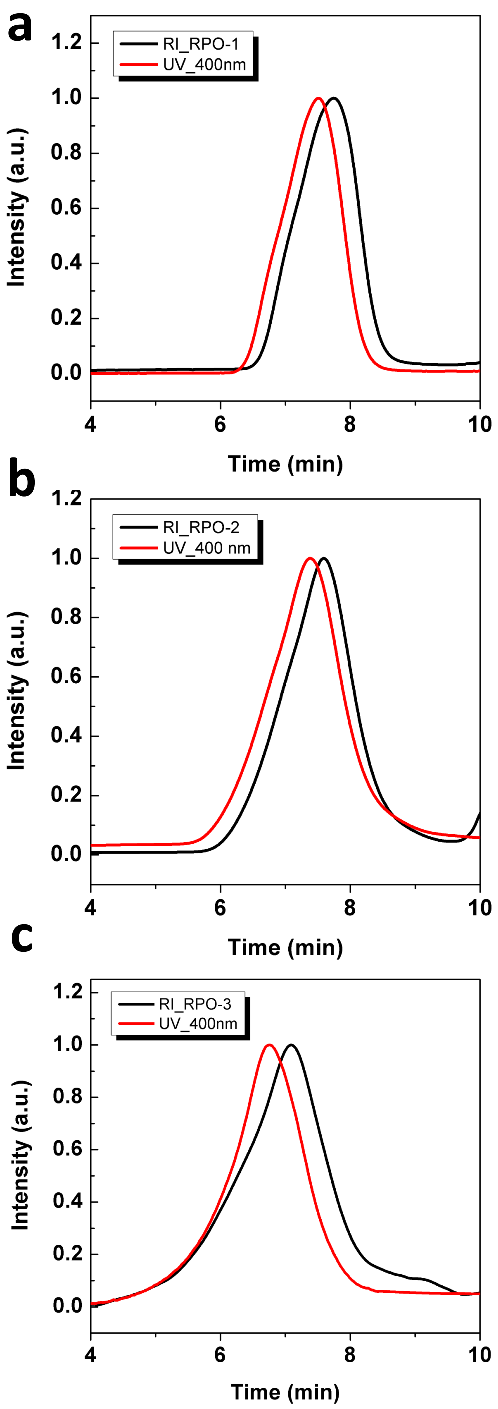

Figure S3. (**a**) GPC traces of RPO-1; (**b**) RPO-2 and (**c**) RPO-3. The shift of the UV traces (red) relative to the refractive index (RI) traces (black) is due to the fact that the eluent flows through the UV-VIS detector first, followed by the RI detector.

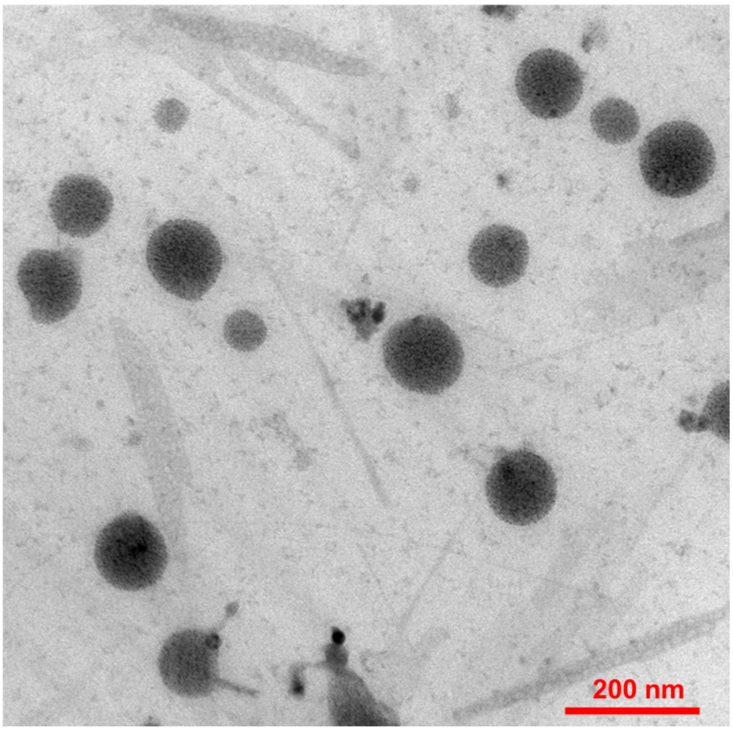

Figure S4. A representative high-magnification TEM image of RPO-1 micellar structures prepared by Method 1.

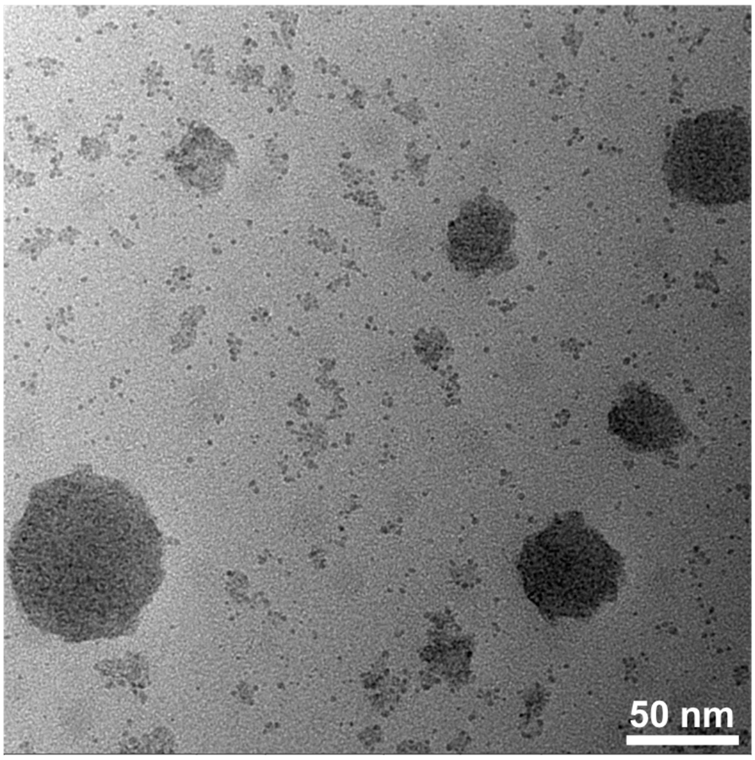

Figure S5. A representative high-magnification TEM image of RPO-1 micellar structures prepared by Method 3.

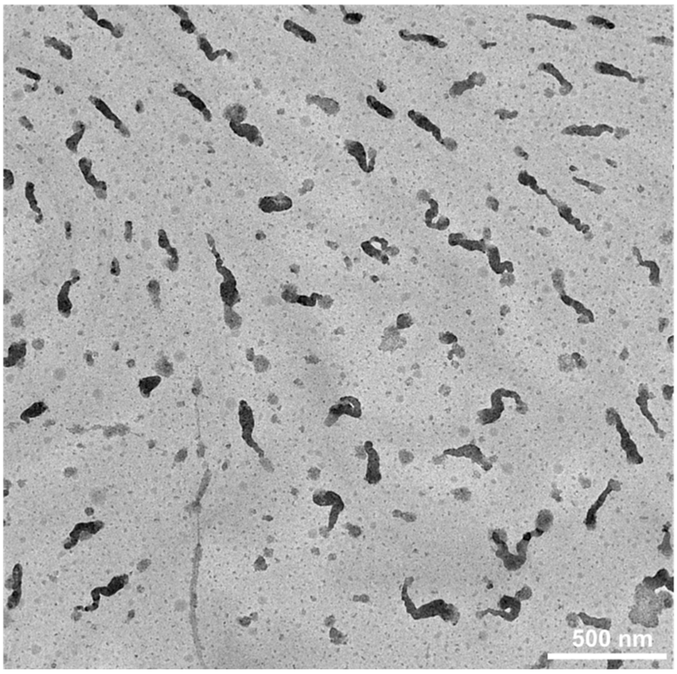

Figure S6. A representative high-magnification TEM image of RPO-1 self-assemblies prepared by Method 4.

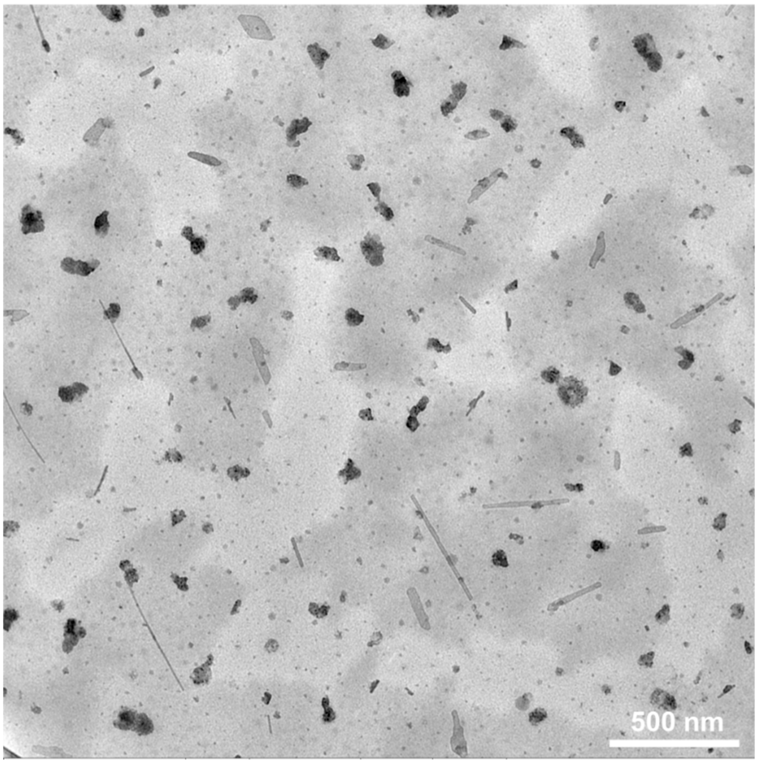

Figure S7. A representative high-magnification TEM image of RPO-2 self-assemblies prepared by Method 4.

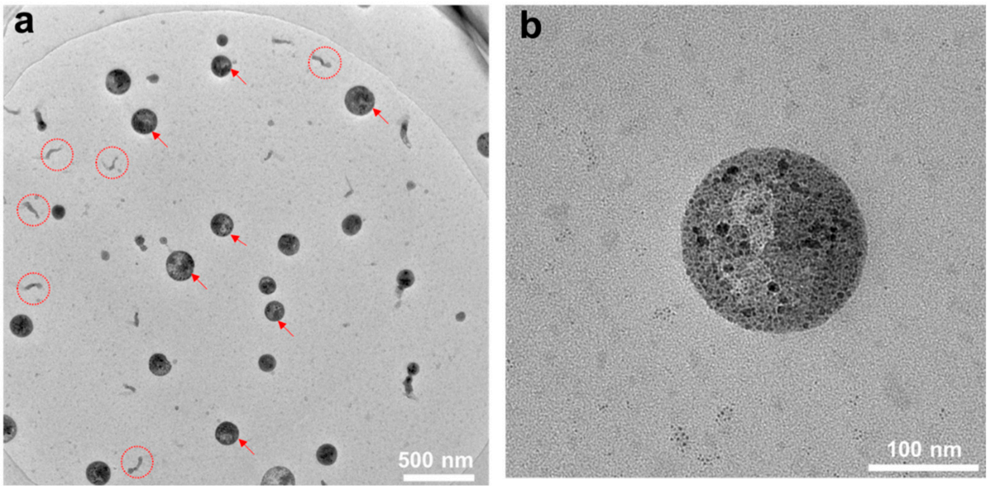

Figure S8. Representative low-magnification (**a**) and high-magnification (**b**) TEM images of RPO-3 self-assemblies prepared by Method 4. Image (**a**) shows the presence of both worm-like micelles (circled by red lines) and large-compound micelles (labeled by red arrows). Image (**b**) highlights the detailed structure of a large compound micelle from image (**a**).

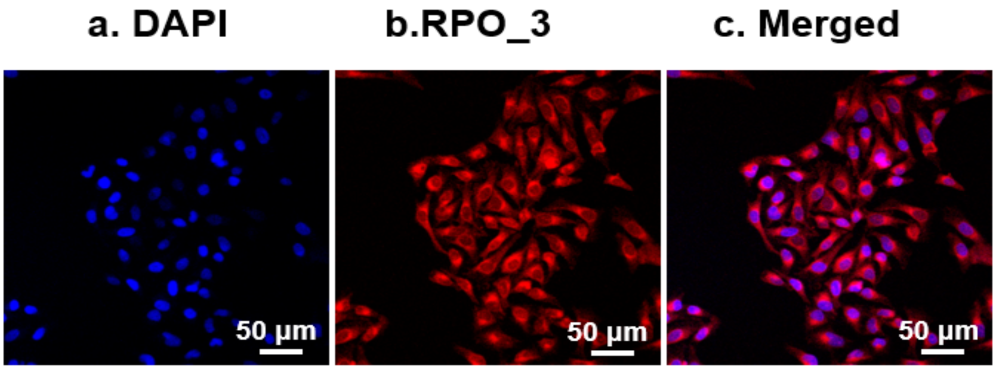

Figure S9. Fluorescence images of HeLa cells after being incubated with blank RPO-3 micelles over 2 h. The fluorescence of DAPI (λ_{ex} = 405 nm) and RPO-3 (λ_{ex} = 488 nm, λ_{em} = 600-700 nm) was pseudo labeled with blue (**a**) and red (**b**), respectively. Image (**c**) is merged from image (**a**) and (**b**).
